# MFHAS1 promotes colorectal cancer progress by regulating polarization of tumor-associated macrophages via STAT6 signaling pathway

**DOI:** 10.18632/oncotarget.12807

**Published:** 2016-10-21

**Authors:** Wankun Chen, Yajun Xu, Jing Zhong, Huihui Wang, Meilin Weng, Qian Cheng, Qichao Wu, Zhirong Sun, Hui Jiang, Minmin Zhu, Yu Ren, Pingbo Xu, Jiawei Chen, Changhong Miao

**Affiliations:** ^1^ Department of Anesthesiology, Fudan University Shanghai Cancer Center, Department of Oncology, Shanghai Medical College, Fudan University, Shanghai, China

**Keywords:** MFHAS1, tumor-associated macrophages, macrophage polarization, colorectal cancer

## Abstract

Malignant fibrous histiocytoma amplified sequence 1 (MFHAS1) is a predicted oncoprotein that demonstrates tumorigenic activity *in vivo*; however, the mechanisms involved are unknown. Macrophages are divided into the pro-inflammatory M1 and anti-inflammatory/protumoral M2 subtypes. Tumor cells can induce M2 polarization of tumor-associated macrophages (TAMs) to promote metastasis; but the underlying pathways require to be elucidated. In this study, we detected a positive association between MFHAS1 expression in TAMs and human colorectal cancer (CRC) TNM stage. Supernatant of CT26 murine CRC cells induced MFHAS1 expression in RAW264.7 murine macrophages. Additionally, CT26 supernatant induced the M2 marker CD206 and activated the pro-M2 STAT6 and KLF4 signaling in control but not MFHAS1-silenced RAW264.7 macrophages. Moreover, supernatant of control, but not MFHAS1-silenced macrophages promoted CT26 cell proliferation, migration and epithelial-mesenchymal transition. Compared with control macrophages, MFHAS1-silenced macrophages showed significantly reduced protumoral effects *in vivo*. Together, these results suggested that CRC cells induce M2 polarization of TAMs through MFHAS1 induction and subsequent STAT6 and KLF4 activation to promote CRC progress. Finally, similar to CT26 supernatant stimulation, peroxisome proliferator-activated receptor-γ (PPARγ) activation by rosiglitazone induced M2 polarization of RAW264.7 macrophages through MFHAS1-dependent pathway. Our results highlight the role of MFHAS1 as a regulator of macrophages polarization and CRC progress.

## INTRODUCTION

Macrophages are critical effectors and regulators of many organ systems and diseases including adaptive immunity, tissue regeneration, hematopoiesis, cardiovascular and metabolic diseases, and cancer [1–5]. In response to physiologic or pathologic microenvironment-derived stimuli, macrophages adopt a spectrum of properties and activation states represented by two main subtypes – the classically activated/inflammatory (M1) and alternatively activated/anti-inflammatory (M2) macrophages [6, 7]. In general, M1 macrophages secret high levels of IL-12, IL-6, TNF-α and low levels of IL-10, and participate in inflammatory response, pathogen clearance, and antitumor immunity. In contrast, M2 macrophages produce high levels of IL-10, TGF-β and low levels of IL-12, and contribute to anti-inflammatory response, wound healing, and protumoral properties [8]. In particular, M2 macrophages have been shown to promote tumor growth, invasion, and metastasis through the secretion of growth factors, matrix metalloproteinases, and the inhibitory cytokines IL-10 and TGF-β to hamper antitumor immunity [9].

Macrophages infiltrating into malignant tumor tissues (the so called tumor-associated macrophages [TAMs]) form the major leukocytic infiltrate of many tumor types. It is generally accepted that most TAMs have the M2-like phenotype [10]. Clinical and experimental evidence has shown that TAMs support tumor growth, invasion and metastasis [11, 12]. Consistent with these functions, a higher density of TAMs, especially the M2-like phenotype, is associated with worse clinical prognosis and/or chemo-resistance in many human cancers [9, 13–15]. Several agents that inhibit TAMs infiltration and/or TAMs polarization into the M2 protumoral phenotype have shown anti-tumor effects in animal models [16], indicating that both TAMs infiltration and TAMs polarization can be considered as a promising target for cancer treatment.

Emerging *in vitro* and *in vivo* evidence has revealed that tumor cells can directly induce TAMs polarization to the M2 phenotype to promote tumor metastasis [17, 18]; however, the underlying molecular mechanisms remain unknown. Malignant fibrous histiocytoma amplified sequence 1 (MFHAS1 or MASL1), a member of the ROCO protein family, is a predicted oncoprotein in malignant fibrous histiocytomas (MFHs), gastrointestinal tumors, and B-cell lymphoma [19–21]. The tumorigenic activity of MFHAS1 has been confirmed in an *in vivo* tumorigenesis assay with nude mice 20, but the underlying mechanisms remain unclear. The aim of this present study is to investigate the relationship of MFHAS1 in colorectal cancer (CRC) cell-induced macrophages polarization and CRC progression.

## RESULTS

### Expression of MFHAS1 in CRC tissues and TAMs is associated with human CRC TNM stage

**Figure 1:**
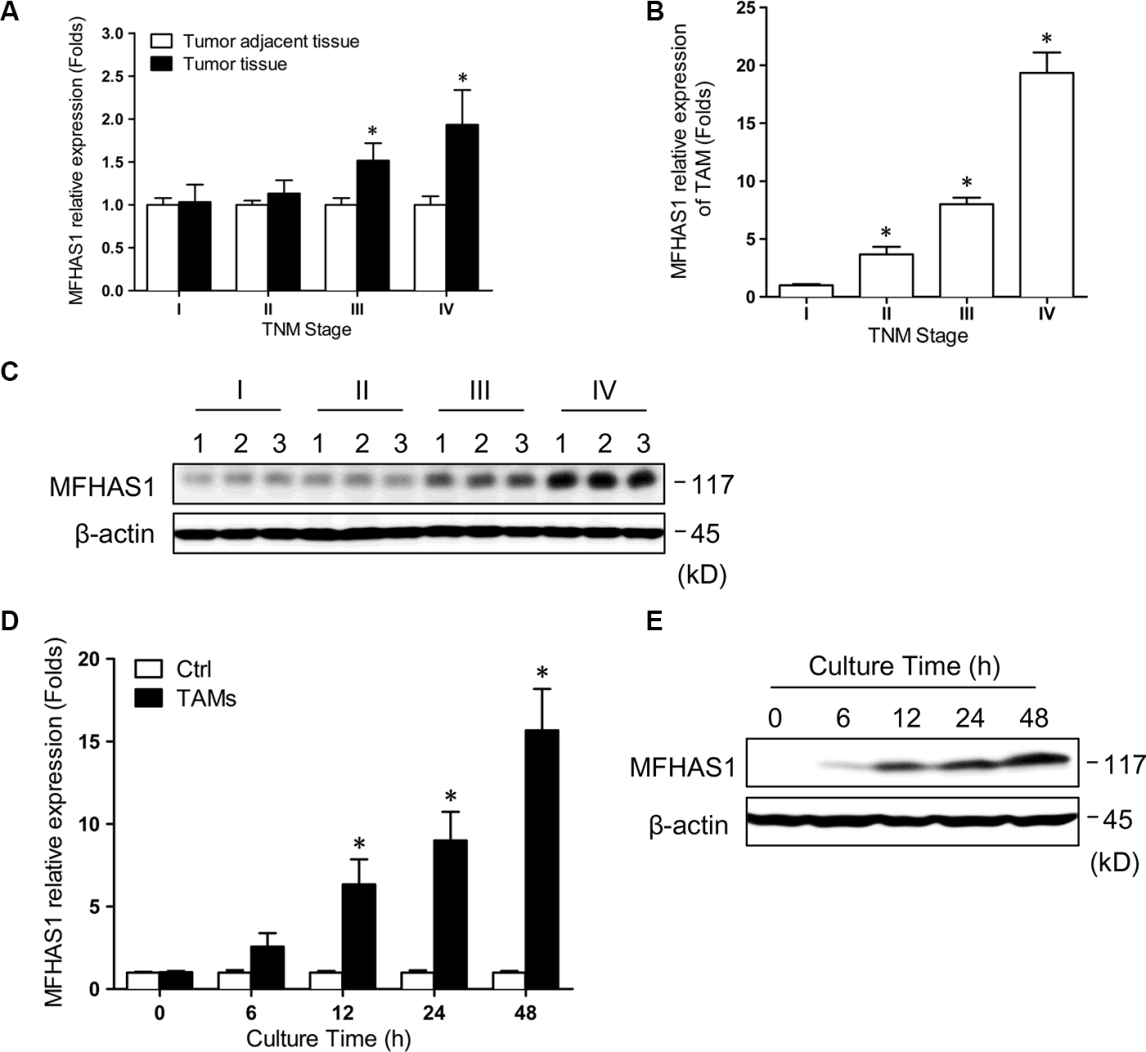
MFHAS1 expression in TAMs is significantly associated with human CRC TNM stage (**A**) MFHAS1 mRNA levels in CRC tumor tissues and tumor adjacent tissues isolated from human CRC tumors samples (TNM stage I – IV) by qRT-PCR. *P < 0.01 vs. grade I. (**B**) MFHAS1 mRNA levels in TAMs isolated from human CRC tumor tissues (TNM stage I – IV) by qRT-PCR. *P < 0.01 vs. grade I. (**C**) MFHAS1 protein levels in TAMs isolated from human CRC tumor tissues (TNM stage I – IV, 3 for each grade) by western blotting. RAW264.7 murine macrophages were incubated with culture supernatant of CT26 murine CRC cells or medium alone for up to 48 h. (**D**) MFHAS1 mRNA expression by qRT-PCR. *n* = 3, *P < 0.01 vs. control. (**E**) MFHAS1 protein expression in cells incubated with CT26 supernatant by western blotting. Ctrl: Group Control.

All of the tumors were confirmed to be adenocarcinomas by postoperative pathological examination. The demographic and clinical characteristics of the patients are shown in Supplementary Table S1. First, we determined the MFHAS1 mRNA levels in CRC tumor tissues and tumor adjacent tissues isolated from human CRC tumors samples of TNM stage I (*n* = 8), II (*n* = 13), III (*n* = 10), and IV (*n* = 7) using qRT-PCR. Our data revealed a positive correlation between the MFHAS1 mRNA level and the CRC TNM stage, with a significantly higher level being detected in CRC cells of grade IV tumors compared with grade I (Figure 1A). Similarly, we determined the MFHAS1 mRNA and protein levels in TAMs isolated from human CRC tumor tissues. MFHAS1 mRNA and protein levels in TAMs increased with the TNM stage as shown in Figure 1B and Figure 1C. These results indicated that MFHAS1 is a potential oncoprotein in human CRC.

### CRC cells induce MFHAS1 expression in macrophages

Since the functions and activation states of TAMs are often shaped by the tumor microenvironment including tumor cells, we speculated that the high MFHAS1 expression in TAMs of high-grade CRC tumors might be attributed to prolonged interaction with tumor cells. To test this hypothesis, we assessed MFHAS1 expression in RAW264.7 murine macrophages incubated with the culture supernatant of CT26 murine CRC cells. Indeed, CT26 supernatant significantly induced MFHAS1 mRNA expression in RAW264.7 macrophages in a time dependent manner (Figure 1D). Western blot analysis of MFHAS1 protein expression generated similar results (Figure 1E). These results suggested that, within CRC tumor tissues, tumor cells could induce MFHAS1 expression in nearby TAMs in a paracrine manner.

### CRC cells induce M2 polarization of macrophages through MFHAS1

**Figure 2:**
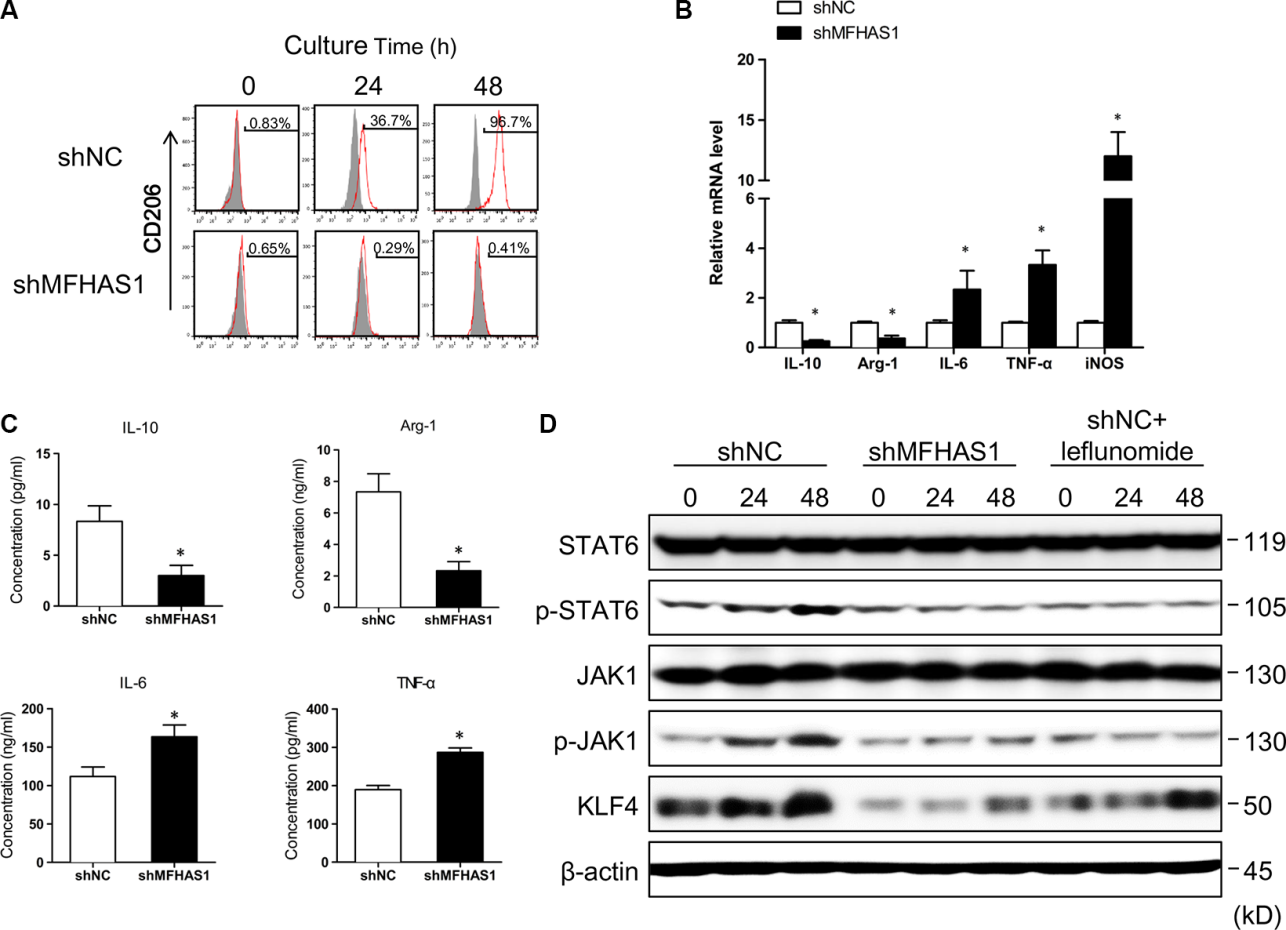
CRC cells induce M2 polarization of macrophages through MFHAS1 (**A**–**C**) RAW264.7 macrophages stably transfected with shMFHAS1 or shNC (a scrambled shRNA, Addgene #1864, as negative control) were incubated with CT26 supernatant for up to 48 h. (A) Surface expression of the M2 marker CD206 after indicated incubation time by flow cytometry. (B) The mRNA expression of the M2 markers IL-10, Arg-1, and MMR and the M1 markers IL-6, TNF-α, and iNOS after 48 h incubation by qRT-PCR. (C) The concentrations of the M2 cytokines IL-10 and Arg-1 and the M1 cytokines IL-6 and TNF-α in the culture media after 48 h incubation by ELISA. *n* = 3, *P* < 0.01vs. shNC. (**D**) RAW264.7 macrophages stably transfected with shMFHAS1 or shNC were incubated with CT26 supernatant in the presence or absence of leflunomide as indicated. The protein levels of JAK1, p-JAK1, STAT6, p-STAT6, and KLF4 before treatment (0) and after 24 h and 48 h treatment were determined by western blotting. *n* = 3, *P* < 0.05 vs. before treatment (0).

To explore the functional significance of this tumor cell-induced MFHAS1 expression, we established RAW264.7 macrophages stably transfected with an MFHAS1-targeting shRNA (shMFHAS1) or a scrambled shRNA (shNC). Compared with shNC-transfected macrophages, shMFHAS1-transfected cells showed significantly suppressed MFHAS1 mRNA and protein expression (Supplementary Figure S1). Our flow cytometric analysis revealed that both shNC and shMFHAS1-transfected RAW264.7 macrophages had very low surface expression of the M2 marker CD206 (Figure 2A); thus most cells were originally in the M1 activation state. Intriguingly, incubating shNC-transfected macrophages with CT26 supernatant led to a time-dependent increase in the surface expression of CD206, suggesting that CT26 supernatant induced polarization of these macrophages to the M2 phenotype (Figure 2A). In contrast, incubating shMFHAS1-transfected macrophages with CT26 supernatant failed to change the CD206 expression of these macrophages (Figure 2A). These data indicated that CT26 cells induce M2 polarization of macrophages through MFHAS1 induction. Consistent with these results, shMFHAS1-transfected macrophages, compared with shNC-transfected cells, showed significantly decreased expression of the M2 markers IL-10, Arg-1, and MMR and increased expression of the M1 markers IL-6, TNF-α and iNOS, along with reduced secretion of the M2 cytokines IL-10 and Arg-1 and increased secretion of the M1 cytokines IL-6 and TNF-α, after treatment with CT26 supernatant (Figure 2B, 2C).

### CRC cells activate STAT6 and KLF4 in macrophages through MFHAS1

The M2 polarization of macrophages is driven and maintained by transcription factors including IRF4, C/EBP-β, Krüppel-like factor 4 (KLF4), STAT6 and peroxisome proliferator-activated receptor-γ (PPARγ) receptor [22]. Specifically, STAT6 and KLF4, both of which induced by IL-4, work cooperatively to drive M2 polarization [23]. In this study, we found that CT26 supernatant induced JAK1 and STAT6 phosphorylation, as well as KLF4 protein expression in shNC- but not shMFHAS1-tranfected RAW264.7 macrophages (Figure 2D). These data suggested that CT26-induced M2 polarization is regulated by STAT6 and KLF4, which are activated following MFHAS1 induction. Moreover, we found that leflunomide, a known JAK/STAT6 inhibitor [24], prevented CT26-induced KLF4 expression (Figure 2D), suggesting that KLF4 was induced by STAT6 under these conditions. These data were in agreement with previous reports that STAT6 and KLF4 induce each other and work cooperatively to drive M2 polarization [23]. Taken together, our results supported that CT26 cells promote M2 polarization of macrophages through induction of MFHAS1 and subsequent activation of STAT6 and KLF4.

### MFHAS1 knockdown in macrophages mitigates CRC progress *in vitro* and *in vivo*

**Figure 3:**
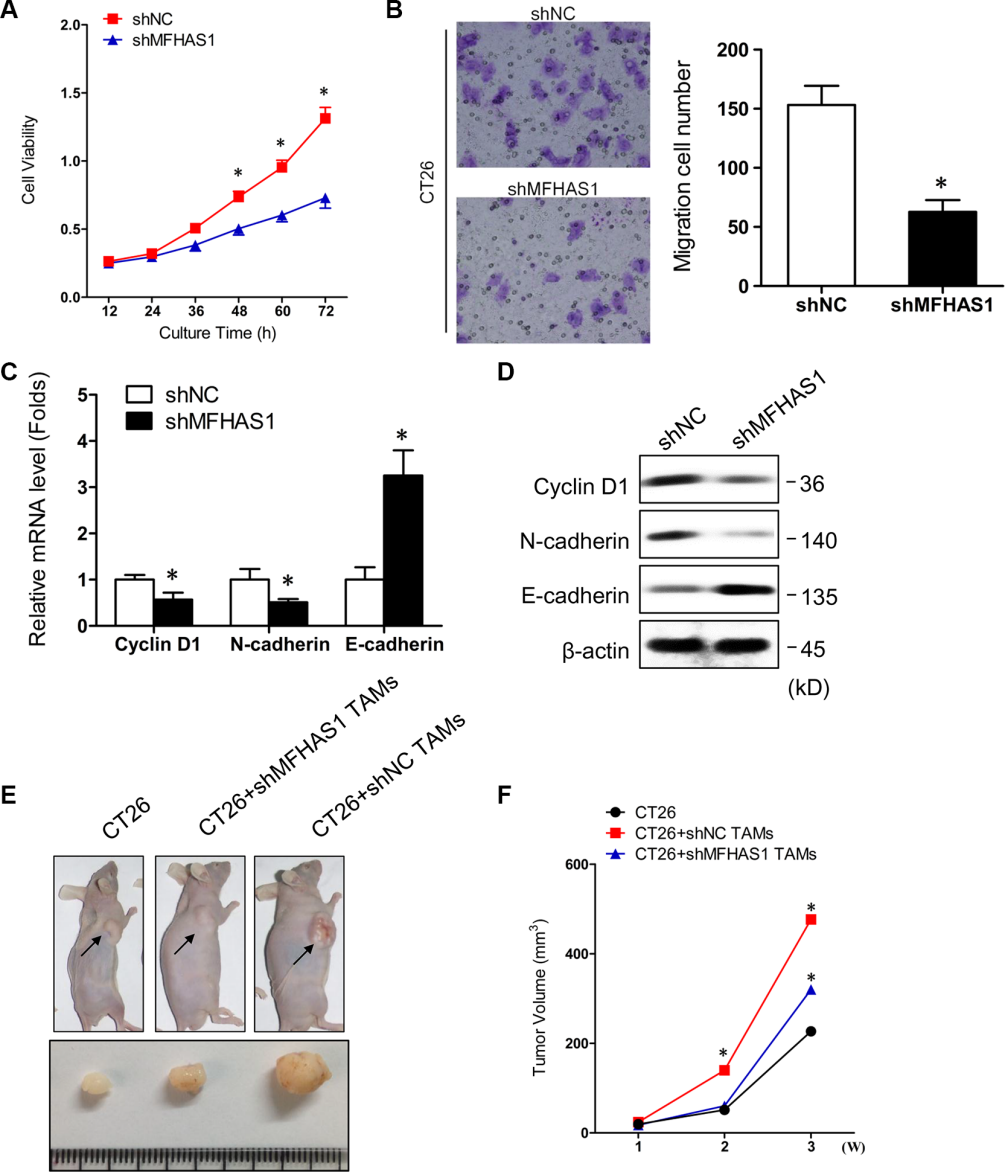
MFHAS1 knockdown in macrophages inhibits CRC *in vitro* and *in vivo* (**A**–**D**) CT26 cells were incubated with culture supernatants of shMFHAS1 or shNC-transfected RAW264.7 macrophages. Cells cultured in control medium without macrophage supernatant were included as control. (A) Cell proliferation by CCK-8 assay. (B) Cell migration by Transwell assay. (C) The mRNA expression of the EMT-related markers Cyclin D1, N-cadherin, and E-cadherin by qRT-PCR. (D) The protein expression of the EMT-related markers Cyclin D1, N-cadherin, and E-cadherin by western blotting. *n* = 3, *P* < 0.05 vs. shNC. (**E**, **F**) CT26 cells together with shMFHAS1 or shNC-transfected RAW264.7 macrophages were inoculated subcutaneously into the right flank of male BALB/c nude mice (*n* = 3 for each group). Mice inoculated with CT26 cells alone were used as control. Mice were sacrificed on day 28. (E) Tumor images on day 28. (F) Tumor volume measured once a week with a caliper. *P* < 0.01 vs. CT26 + shNC RAW264.7.

Previous studies have reported that tumor cells can regulate macrophage polarization, and in return, the tumor cell-educated macrophages can impact tumor metastasis *in vitro* and *in vivo* [18, 25]. In this study, we found that, incubating CT26 cells with supernatant of shNC-transfected RAW264.7 macrophages resulted in faster cell proliferation and migration (Figure 3A, 3B), as well as decreased expression of the epithelial marker E-cadherin and increased expression of the mesenchymal marker Cyclin D1 and N-cadherin (Figure 3C, 3D). These data indicated that macrophage-derived factors stimulate CRC cell growth, migration, and epithelial-mesenchymal transition (EMT) *in vitro*. In contrast, supernatant of shMFHAS1-transfected RAW264.7 macrophages failed to promote CT26 cell growth, migration, and EMT (Figure 3A – 3D). Since M2 but not M1 macrophages exhibit protumoral properties, the lack of pro-CRC effects of MFHAS1-silenced macrophages was presumably attributed to inhibited M2 polarization of these cells.

To find out whether our *in vitro* data can be extrapolated to *in vivo* situation, we established a mouse CRC xenograft model. Male BALB/c nude mice were subcutaneously inoculated with CT26 cells together with shMFHAS1 or shNC-transfected RAW264.7 macrophages (CT26 + shMFHAS1 RAW264.7 and CT26 + shNC RAW264.7, respectively). Mice inoculated with CT26 cells alone were included as control. The tumors were harvested on day 28. Compared with control, mice from the CT26 + shNC RAW264.7 group showed significantly accelerated tumor growth (Figure 3E, 3F), demonstrating that TAMs promote CRC tumorigenesis *in vivo*. Consistent with our *in vitro* data, tumor growth in the CT26 + shMFHAS1 RAW264.7 group was faster than control but slower than the CT26 + shNC RAW264.7 group (Figure 3E, 3F). The reduced pro-CRC activity of MFHAS1-silenced macrophages *in vivo* was presumably attributed to curtailed M2 polarization of these cells *in vivo*. Thus, MFHAS1 inhibition might provide therapeutic benefits for CRC through regulation of TAMs polarization.

### PPARγ regulates macrophages polarization through induction of MFHAS1

**Figure 4:**
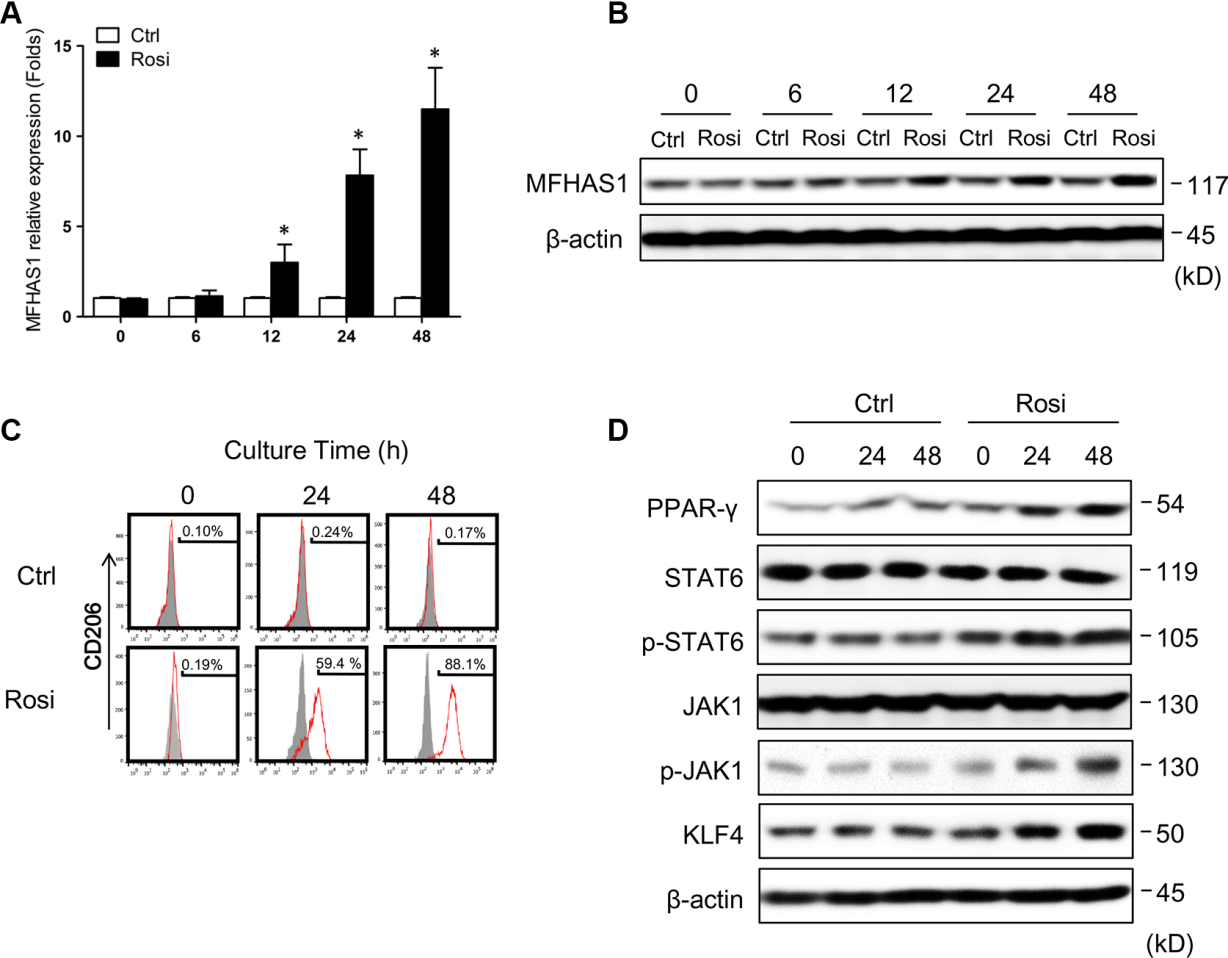
PPARγ regulates macrophage polarization through MFHAS1 RAW264.7 macrophages were treated with the PPARγ agonist rosiglitazone or vehicle alone (control) for up to 48 h. (**A**, **B**) The mRNA (A) and protein expression (B) of MFHAS1 after indicated treatment time by qRT-PCR and western blotting, respectively. *n* = 3, *P* < 0.05 vs. control. (**C**) Surface expression of the M2 marker CD206 after indicated treatment time by flow cytometry. (**D**) The protein levels of PPARγ, JAK1, p-JAK1, STAT6, p-STAT6, and KLF4 after indicated treatment time by western blotting. Rosi: rosiglitazone.

PPAR (peroxisome proliferator-activated receptor) γ is rapidly induced upon differentiation of monocytes into macrophages [26], and promes primary monocytes into M2 differentiation [27]. In addition, recent studies have shown that ovarian cancer stem cells induce M2 polarization of macrophages by activating PPARγ and NF-κB [28]. We found that PPARγ is a potential regulatory element for MFHAS1 gene in DECODE database of SABiosciences (Qiagen), so we hypothesized that the PPARγ in CRC microenviroment induced the express of MFHAS1 in TAMs. In this study, we found that the PPARγ agonist rosiglitazone [29] induced MFHAS1 and CD206 expression in RAW264.7 macrophages (Figure 4A–4C), and specifically, these changes were accompanied by increased JAK1/STAT6 phosphorylation and KLF4 expression (Figure 4D). These data suggested that, similar to CT26 supernatant stimulation, PPARγ activation drives M2 polarization of macrophages through MFHAS1-mediated activation of STAT6 and KLF4 signaling.

## DISCUSSION

In this study, we found that MFHAS1 expression in TAMs isolated from CRC tissues was positively associated with the human colorectal cancer (CRC) TNM stage. Our *in vitro* studies revealed that culture supernatant of CT26 murine CRC cells induced M2 polarization of RAW264.7 murine macrophages through induction of MFHAS1 and subsequent activation of pro-M2 STAT6 and KLF4 signaling. MFHAS1 knockdown reduced the protumoral effects of RAW264.7 macrophages on CT26 cells *in vitro* and *in vivo*. Furthermore, similar to CT26 supernatant stimulation, PPARγ activation by rosiglitazone induced M2 polarization of RAW264.7 macrophages through activation of MFHAS1, STAT6 and KLF4. These results highlighted the role of MFHAS1 in CRC tumor cell-induced macrophages polarization and CRC metastasis.

During tumor progression, circulating monocytes and macrophages were actively recruited into tumors. These TAMs closely resemble the M2-polarized macrophages, and have been shown to stimulate tumor growth, invasion, and metastasis [30]. Extensive *in vitro* and *in vivo* experiments have demonstrated that targeting TAMs, either their recruitment into tumors or M1/M2 functional switch, may provide therapeutic benefits for cancer [16, 31]. Moreover, several recent studies have shown that the anticancer effects of a number of known chemotherapeutic agents are accredited to cytotoxicity against TAMs and/or inhibition of M2 macrophage polarization [32–35]. These data provided strong support for TAM-targeting therapeutics for cancer.

Within tumor tissues, macrophages M1/M2 polarization is regulated by various microenvironmental signals derived from tumor and stromal cells. In particular, tumor cells have been shown to directly induce M2 macrophage polarization to promote tumorigenesis [17, 18]. In *in vitro* coculture systems with adult T-cell leukemia/lymphoma (ATLL) cells and macrophages, the M2 macrophage marker CD163 was strongly induced by direct contact with ATLL cells, and CD163 knockdown in macrophages significantly suppressed ATLL cell growth [17]. Moreover, placental growth factor (PLGF) secreted by larynx carcinoma (LC) cells triggered M2 polarization of macrophages via transforming growth factor (TGF) β receptor activation, and thereby promoted LC growth [18]. In this study, we found that MFHAS1 expression in TAMs was positively associated with human CRC TNM stage. CRC cell supernatant induced the M2 marker CD206 in macrophages through induction of MFHAS1 and subsequent activation of JAK1/STAT6 and KLF4. MFHAS1 knockdown in macrophages reduced M2 polarization and inhibited CRC cell growth, migration, and EMT *in vitro* and tumor formation *in vivo*. These results highlighted the role of MFHAS1 in the crosstalk between CRC tumor cells and TAMs, which activates TAMs polarization and CRC progression.

There is limited information on the function and mechanisms of MFHAS1. Although the tumorigenic activity of MFHAS1 has been reported in an *in vivo* tumorigenesis assay [20], the mechanisms involved have never been reported. Interestingly, MFHAS1 has been identified as a pathogen-responsive gene in human primary macrophages, where it regulates Toll-like receptor (TLR)-dependent signaling [36, 37]. Specifically, MFHAS1 knockdown in RAW264.7 macrophages enhanced IL-6 production following LPS stimulation, suggesting an anti-inflammatory function for MFHAS1 [36, 37]. In this study, we found that MFHAS1 knockdown in RAW264.7 macrophages significantly increased IL-6 mRNA expression and protein secretion after CT26 supernatant treatment. MFHAS1 induced by CRC cell supernatant drives macrophages polarization to the anti-inflammatory M2 phenotype. These findings were in agreement with the immune modulatory function of MFHAS1 reported in previous studies [36, 37]. Whether the MFHAS1 amplification in CRC tissues is from gene duplication or only from the transcription require further gene sequencing.

Macrophage polarization is regulated by multiple interacting endogenous cellular mechanisms. Our results showed that PPARγ drives M2 polarization of macrophages through MFHAS1-dependent activation of STAT6 and KLF4. Some studies demonstrated that PPARγ and its agonist rosiglitazone may have opposing effects on tumor progression, with anti-tumorigenic effects on cancer cells, but pro-tumorigenic effects on cells of the microenvironment [38, 39]. Whether MFHAS1 is involved in other signaling pathways that regulate macrophage polarization require further investigation.

## MATERIALS AND METHODS

### Human CRC tissues and mouse CRC and macrophage cell lines

The tumor tissues were collected from a total of 38 CRC patients who underwent surgery at Fudan University Shanghai Cancer Center between June 2014 and December 2014. The study protocol was approved by the Ethics Committee at Fudan University Shanghai Cancer Center. All study participants gave written informed consent. The mouse CRC cell line CT26 and macrophage cell line RAW264.7 were ere provided by Dr. Miao (Fudan University Shanghai Cancer Center, China) as a gift. All cells were cultured in high-glucose DMEM (HyClone, Thermo, USA) supplemented with penicillin, streptomycin, and 10% FBS (HyClone) at 37°C, 5% CO_2_ in a humidified incubator.

### TAM isolation and culture

The fresh CRC tissues were cut into pieces and digested in collagenase B (1 mg/ml, #11088807001, Roche) containing Buffer A. The dissociated cells were collected into a 15 ml tube and centrifuged at 400 g for 5 min. TAMs were isolated from the pellets using a Percoll Density Gradient Centrifugation kit (Pharmacia) following manufacturer's instructions.

### RNA extraction and qRT-PCR

Total RNA was extracted using the TRIzol reagent (Invitrogen). Complementary DNA (cDNA) was synthesized using the PrimerScriptRT Reagent (TaKaRa, Tokyo, Japan) following manufacturer's instructions. The real-time PCR (qRT-PCR) was carried out using a two-step SYBR Green II reaction mix (Applied Biosystems, USA) on an ABI 7500 Real-Time PCR System (Applied Biosystems). The PCR primers used in this study can be found in Supplementary Table S2.

### Western blotting

Samples containing equal amounts of protein were fractionated on a 10% SDS-PAGE gel and transferred onto a Hybond TM-P membrane (GE Healthcare, Little Chalfont, UK) by using Trans-Blot cell (Bio-Rad Laboratories, Hercules, CA, USA). After blocking solution (8% skim milk in TBS-T, according to vendor's suggestion) at room temperature for 1 h, the membranes were incubated with specific antibodies against MFHAS1, p-STAT6, STAT6, PPAR-γ (Santa Cruz Biotechnology, Dallas, TX, USA), p-JAK1, JAK1, Cyclin D1, N-cadherin, E-cadherin, β-actin (Cell Signalling Technology, Danvers, MA, USA) and KLF4 (Abcam, Cambridge, MA, USA), respectively, at 4°C overnight. After washing, the membranes were incubated with horseradish peroxidase (HRP)-conjugated secondary antibodies at room temperature for 1 h. Protein bands were detected by enhanced chemoluminescence.

### Stable MFHAS1 knockdown in RAW264.7 cells

The small hairpin RNA (shRNA) targeting mouse MFHAS1 (shMFHAS1, CCGGCTGAGCAG TTGCAGATTGAATCTCGAGATTCAATCTGCAACTG CTCAGTTTTTG) and a scrambled shRNA used as negative control (shNC, Addgene #1864) were synthesized and cloned into the pLenti-C-Myc-DDK vector (Origene, China) to generate the lentirival expression vectors. For lentivirus production, 1 μg of shRNA expression plasmid together with 1 μg of helper plasmids (0.4 μg pMD2G and 0.6 μg psPAX2) were transfected into 293T cells (ATCC) with Effectene reagent (Qiagen). Viral supernatants were collected 48 h after transfection and cleared through a 0.45-μm filter. RAW264.7 cells were infected with viral supernatants containing 4 μg/ml polybrene (Sigma) for 24 h. After the transduction, the cells were selected with puromycin for 7 days for stable shRNA expression.

### Flow cytometry

ShMFHAS1 or shNC-transfected RAW264.7 cells were incubated with CT26 cell supernatant for up to 48 h. AT specific time points, the cells were collected, incubated with anti-CD206 antibody (Santa Cruz Biotechnology, Dallas, TX, USA) followed by PE-conjugated secondary antibody, and subjected to analysis on a BD FACSCalibur flow cytometer.

### Enzyme-linked immunosorbent assay (ELISA)

ShMFHAS1 or shNC-transfected RAW264.7 cells were incubated with CT26 cell supernatant for 48 h. The concentrations of IL-10, Arg-1, IL-6, and TNF-α in the culture supernatant were determined using Quantikine Kits from R&D Systems (USA) following manufacturer's instructions. Each experiment was conducted in triplicate.

### Cell counting Kit-8 (CCK-8) assay

ShMFHAS1 or shNC-transfected RAW264.7 cells were incubated with CT26 cell supernatant for 24 h. The supernatant was removed and the RAW264.7 cells were cultured with fresh medium for 24 h. CT26 cells were plated in 96-well plates (1 × 10^4^ cells/well) and cultured in 100 μl culture supernatants of shMFHAS1 or shNC-transfected RAW264.7 macrophages. At specific time points, Cell Counting Kit-8 (CCK8, Dojindo, Kumamoto, Japan) was added, and the cells were incubated for another 4 h. The absorbances (optical densities) were recorded with a universal microplate reader (Bio-Tek) at 450 nm. Each experiment was conducted in triplicates.

### Cell migration assay

Cell migration was assessed using a Transwell chamber (Corning). Briefly, CT26 cells were suspended in culture supernatants of shMFHAS1 or shNC-transfected RAW264.7 macrophages. Cells were then loaded to the transwell upper chamber. The medium containing 10% FBS was loaded to the lower chamber. Cells were allowed to migrate for 12 h at 37°C. Non-migrating cells on the upper surface of the membrane were gently removed. The cells that had migrated to the lower surface of the membrane were fixed with methanol, stained with crystal violet, and counted in five randomly selected fields under an inverted light microscope. Each experiment was conducted in triplicate.

### Tumorigenesis in nude mice

Male BALB/c nude mice (4-week old) were purchased from Institute of Zoology, Chinese Academy of Sciences (Shanghai). CT26 cells (1 × 10^6^) together with shMFHAS1 or shNC-transfected RAW264.7 cells (5 × 10^5^) were subcutaneously inoculated into the right flank of mice (*n* = 3 for each group). Mice inoculated with CT26 cells alone were used as control. Tumor nodules were measured once a week with a caliper. Mice were sacrificed on day 28. Tumors were harvested and photographed.

### Statistics

All data were analyzed using SPSS15.0. Results are presented as mean ± SD (standard deviation). Data from different groups were compared using the Student's *t*-test or non-parametric test (Mann-Whitney *U*-test). Differences with a *P* < 0.05 were considered statistically significant.

## SUPPLEMENTARY MATERIALS


