# Supplementary material for: MFHAS1 promotes colorectal cancer progress by regulating polarization of tumor-associated macrophages via STAT6 signaling pathway
Source: Oncotarget. 2016 Oct 21;7(48):78726–35. doi: 10.18632/oncotarget.12807 (PMC5346672; doi:10.18632/oncotarget.12807)
Supplement: Supplementary file 1 [file oncotarget-07-78726-s001.pdf]

MFHAS1 promotes colorectal cancer progress by regulating polarization of tumor-associated macrophages via STAT6 signaling pathway

Supplementary Materials

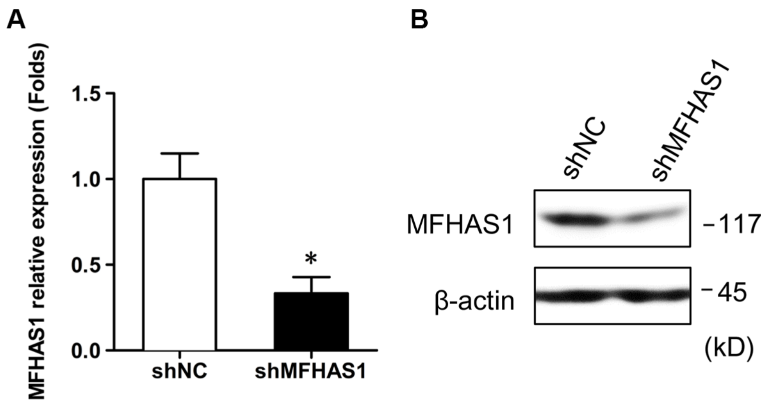

Supplementary Figure S1: RAW264.7 macrophages were stably transfected with shMFHAS1 or shNC. The MFHAS1 mRNA (**A**) and protein expression (**B**) were determined by qRT-PCR and western blotting, respectively.

Supplementary Table S1: The demographic and clinical characteristics of the patients

	TNM stage			
	**I (*n* = 8)**	**II (*n* = 13)**	**III (*n* = 10)**	**IV (*n* = 7)**
Age (year, Mean ± SD)	60.1 ± 9.1	59.7 ± 9.5	59.3 ± 9.2	58.6 ± 7.2
Gender (Male/Female)	5/3	8/5	6/4	4/3

Supplementary Table S2: Primers used in this study

Genes	Sequence (5′–3′)
huMFHAS1-F	GCCTCATGCTAGACAACAACG
huMFHAS1-R	GCTGGTTGCGACTAAGGTAGAG
huGAPDH-F	ACAACTTTGGTATCGTGGAAGG
huGAPDH-R	GCCATCACGCCACAGTTTC
mMfhas1-F	GAGATCATCTGCCCCAAGAA
mMfhas1-R	TTCTGTGCTTCTCACCAACG
mβ-actin-F	CGGTTCCGATGCCCTGAGGCTCTT
mβ-actin-R	CGTCACACTTCATGATGGAATTGA
mIL-10-F	CCAGTTTTACCTGGTAGAAGTGATG
mIL-10-R	TGTCTAGGTCCTGGAGTCCAGCAGACTC
mArg-1-F	GCTCAGGTGAATCGGCCTTTT
mArg-1-R	TGGCTTGCGAGACGTAGAC
mMMR -F	GCAAATGGAGCCGTCTGTGC
mMMR -R	CTCGTGGATCTCCGTGACAC
mIL-6-F	CCATCCAGTTGCCTTCTTGG
mIL-6-R	TTTCTGCAAGTGCATCATCG
mTNF-α-F	CTGTAGCCCACGTCG
mTNF-α-R	TTGAGATCCATGCCGTTG
miNOS -F	GTTTCTGGCAGCAGCGGCTC
miNOS -R	GCTCCTCGCTCAAGTTCAGC
mCyclin D1-F	TGCCATCCATGCGGAAA
mCyclin D1R	AGCGGGAAGAACTCCTCTTC
mE-cadherin-F	CACCTGGAGAGAGGCCATGT
mE-cadherin-R	TGGGAAACATGAGCAGCTCT
mN-cadherin-F	AGGGTGGACGTCATTGTAGC
mN-cadherin-R	CTGTTGGGGTCTGTCAGGAT
